# Commentary: Programmable base editing of A·T to G·C in genomic DNA without DNA cleavage

**DOI:** 10.3389/fgene.2018.00021

**Published:** 2018-02-07

**Authors:** Ianis G. Matsoukas

**Affiliations:** Faculty of Health and Human Sciences, School of Sport and Biomedical Sciences. University of Bolton, Bolton, United Kingdom

**Keywords:** base editing, CRISPR, Cas-9, genome engineering, targeted gene repair

An article recently published in Nature (Gaudelli et al., 2017) reports an approach of altering DNA sequences without cleaving the DNA strands. This method of gene editing, exploits a modified version of the CRISPR-Cas9 system and an RNA-based deamination enzyme.

## Introduction: genome-editing biotechnologies

The clustered regularly interspaced short palindromic repeat (CRISPR) technology is widely used to mediate genome-editing in a variety of species (Sander and Joung, 2014; Barrangou and Doudna, 2016). CRISPR, a microbial cellular immunity system (Barrangou et al., 2007), allows the precise editing of DNA sequences and interrogation of regulatory elements, gene function, and protein networks (Doudna and Charpentier, 2014; Zhang et al., 2014; Amitai and Sorek, 2016). This function requires the presence of a set of CRISPR-associated (Cas) genes, which usually are found adjacent to the CRISPR locus. The wild-type Cas9 endonuclease and its different variants (Jinek et al., 2012; Cong et al., 2013; Qi et al., 2013) have been heavily utilized as the major component in genome-editing protocols. CRISPR relies on the ability of CRISPR single guide RNAs (sgRNAs) to target the Cas9 endonuclease to precise genomic locations, where Cas9 introduces DNA double-strand breaks (Hsu et al., 2013; Doudna and Charpentier, 2014; DSBs). Subsequently, the cell's DNA repair machinery mends the DSBs, commonly resulting in random insertions or deletions (indels) of nucleotides at the location of DSBs (Lieber, 2008).

Several review articles have been published recently on the different classes of customizable DNA-binding endonucleases that can be exploited to manipulate virtually any genomic sequence (Cox et al., 2015; Gaj et al., 2016; Yin et al., 2017). Hence, the genome-editing technologies will not be described in great detail here. Apart from the CRISPR technology (Cong et al., 2013; Mali et al., 2013), to achieve effective genome-editing by inducing site-specific DNA DSBs three other major classes of targeted nucleases are currently being exploited: meganucleases (Smith et al., 2006), zinc finger nucleases (Urnov et al., 2005; Miller et al., 2007), and the transcription activator-like effectors (Boch et al., 2009; Moscou and Bogdanove, 2009; Christian et al., 2010; Miller et al., 2011). However, the ease of use and versatility of CRISPR–Cas system has led to its expeditious and wide adoption for precise modification of DNA sequences.

## Base editing: expanding the genome engineering toolbox

At the genomic level, there are two main types of point mutational changes: nucleotide substitutions and nucleotide additions or deletions. Although some well-known inherited genetic disorders are caused by point mutations (Bamshad et al., 2011; Gilissen et al., 2011; Veltman and Brunner, 2012), current genome-editing technologies to point mutation correction are unsuitable. The correction rates of the conventional genome-editing technologies are 0.1–5%, and typically they induce a plethora of random indels at the target locus resulting from the cellular reaction to DSBs (Cox et al., 2015; Hilton and Gersbach, 2015). It has been proposed that these indels might lead to side effects in therapeutic applications.

When the repair, small alteration or introduction of a point mutation at a specific genomic site is desired rather than stochastic disruption of the whole locus, base editing, a novel genome-editing toolbox offers a more efficient strategy. Base editing allows the direct, irreversible chemical conversion of a specific DNA base pair to a different base pair at a target genomic locus without requiring DSBs, homology directed repair processes, or donor DNA templates (Komor et al., 2016, 2017; Nishida et al., 2016). Hence, base editing is an exciting recent addition to the genome-editing toolbox.

## Refining CRISPR for point mutation repair

The ACTIVATION INDUCED DEAMINASE (AID)/APOLIPOPROTEIN B mRNA EDITING ENZYME, CATALYTIC POLYPEPTIDE-LIKE (APOBEC) family comprises of proteins with diverse physiological functions. The family represents a group of cytidine deaminases that enzymatically deaminate deoxycytidine (C) to deoxyuridine (U) in single-stranded DNA sequences. AID is one of the well-studied members of the APOBEC family (Knisbacher et al., 2016; Salter et al., 2016).

Most of the human pathogenic point mutations have been attributed to cytosine/guanine (C/G)→ thymine/adenine (T/A) base pair transitions. Interestingly, Komor et al. (2016) created several base editors (BEs) that convert C/G→ A/T in specific-sequence contexts. They achieved this by combining three polypeptides: (i) a cytidine deaminase; (ii) a mutated Cas9 CRISPR protein that doesn't cleave DNA but uses an associated sgRNA to target specific genomic sequences; and (iii) a protein that prevents reversion of U→ C. After the cytidine deaminase changes C→ U, the BE “nicks” the strand opposite the modification site to induce the cellular apparatus to replace G→ A and change U→ T. Among the different APOBEC enzymes exploited, the rAPOBEC1 showed the highest deaminase activity. Interestingly, the fact that different members of the AID/APOBEC family have different specificity (Kouno et al., 2017), this may allow the targeting of different bases.

The recent publication by Gaudelli et al. (2017) describes the structure and function of a novel base editing tool, the adenine base editor (ABE). Interestingly, the ABE can accomplish the opposite function: the A/T→ G/C transition (Gaudelli et al., 2017). Several generations of ABE have been created (Gaudelli et al., 2017). The 7th generation of ABE (e.g., ABE7.10) utilizes a version of the CRISPR-Cas9 system which has the cleavage ability deactivated (Gaudelli et al., 2017). As a result, ABEs are able to specifically alter base pairs in the genome without having to rely on unpredictable repair systems to introduce indels for gene deactivation, or to splice in new loci.

**Figure 1:**
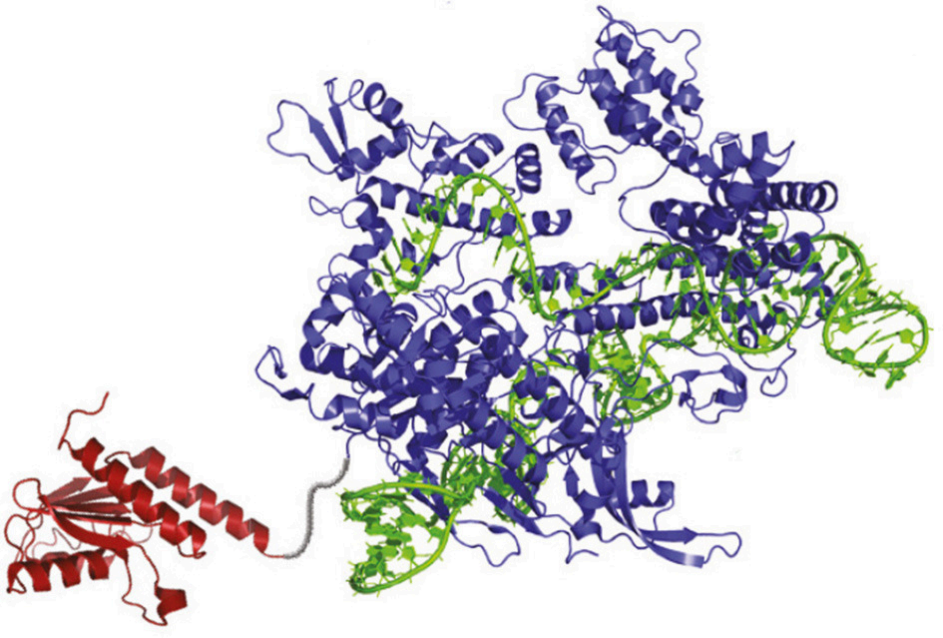
The Structure of the Adenine Base Editor (ABE). The ABE contains an atom-rearranging enzyme (in red) that can convert adenine to inosine (read and copied as guanine), sgRNA (in green), which directs the molecule to the desired point, and a Cas9 nickase (in blue), which snips the opposing strand of DNA and tricks the cell into converting the complementary base (Gaudelli et al., 2017).

The development of the ABEs is one of the most exciting developments in genome-editing technologies. The ABEs are composed of three main components: an “evolved” version of transfer RNA adenosine deaminase (TadA), a Cas9 “nickase,” and a sgRNA (Figure 1; Gaudelli et al., 2017). TadA is the starting point for ABEs due to their property to convert A→ inosine (I) in the single-stranded anticodon loop of tRNA^Arg^, which the cells treat as G (Kim et al., 2006). The same conversion can also be carried out by the ADENOSINE DEAMINASES THAT ACT ON RNA (ADARs) enzymes (Zheng et al., 2017). It seems that TadA was selected as an approach due to the fact that, unlike ADAR, TadA does not require small-molecule activators (Macbeth et al., 2005). Natural TadA, however, works only on transfer RNA, not DNA. To create DNA-modifying versions of TadA, Gaudelli et al. (2017) generated TadA mutants in *Escherichia coli* S103041 strain. The *E. coli* mutant lines were programmed to convert A→ I in linkage to antibiotic resistance genes in order to survive the antibiotic challenge. The surviving *E. coli* encoded TadA mutations imparted the ability to perform the A→ I conversion on DNA.

To assemble the ABE, Gaudelli et al. (2017) attached a mutated TadA to a modified form of the Cas9 enzyme. Ordinarily, as part of a CRISPR/Cas9 gene-editing system, Cas9 cleave DNA, which is then repaired by one or another set of molecular apparatuses residing in the cell. However, the mutated “nickase” version of the Cas9 enzyme that has been exploited by Gaudelli et al. (2017) induces a single-strand DNA break (nick) at a specific point based on the co-expressed sgRNA-defined target sequence, rather than a DSB conferred by the native enzyme. Hence, the Cas9 exploited in ABE only nicks DNA at the base opposite the A→ I conversion site. The “nick” prompts the cell to insert the correct partner base pair to match the new one, thereby completing the transition of A/T→ G/C. This specific property of the ABE has led Gaudelli et al. (2017) referring to ABE as a gene-editing “pencil,” “overwriting errors” in the genomic sequence, instead of the “scissors” that CRISPR technology is usually compared to.

Working with samples taken from patients, Gaudelli et al. (2017) continued the work of Komor et al. (2016), by exploiting the novel ABE tool to repair a point mutation that confer hereditary hemochromatosis, a pathological phenotype characterized by excessive intestinal absorption of dietary iron that leads to excessive iron uptake and deposition in organs (Alexander and Kowdley, 2009). In addition, Gaudelli et al. (2017) exploited the ABE in human cells lines to induce a mutation that overcomes sickle-cell anemia (Traxler et al., 2016). Interestingly, in both experimental approaches, they detected virtually no off-target effects, or indels (typically ≤ 0.1%), which are a concern with the conventional approaches of implementing CRISPR to modify whole loci.

## Concluding remarks

The development of ABEs by Gaudelli et al. (2017) is a significant addition to the genome-engineering toolbox. BEs use a component of CRISPR, but they have some advantages over the standard CRISPR technology. CRISPR is ideal for inserting and deleting DNA sequences at targeted locations in a genome. But BEs have the edge for single-base modifications because they are significantly more efficient than standard CRISPR at making single-base substitutions. However, BEs are not meant to be a replacement to conventional genome-editing with CRISPR. But it is rather an additional tool for modifying the genome in an attempt to repair flawed genes in cells that cause diseases.

ABEs greatly expand the scope of base editing. Together with previously described base editors, the work of Gaudelli et al. (2017) enable the programmable installation of all four transitions (C→ T, A→ G, T→ C, and G→ A) in genomic sequences. Hence, ABEs induce point mutations more efficiently and systematically than a current Cas9 endonuclease-based biotechnologies, induce less off-target genome edits than Cas9, and can induce disease-repairing or disease-suppressing mutations in human genome. Since many genetic diseases arise from point mutations, BEs such as the ABE, have significant implications in the elucidation of molecular genetic mechanisms of cells in health and disease states.

However, more work remains before BEs can be used to treat patients with genetic diseases. This includes tests of safety, efficacy, side effects, and advances in methods to deliver these large biological molecules involved to the diseased tissues. In addition, the strategies developed and implemented by Gaudelli et al. (2017) expand the utility and applicability of BEs. However, additional research is required on improving the function of sgRNAs to target diverse genomic sites. Due to the presence of repetitive DNA elements in the genome and the requirements for specific parameters in designing sgRNAs, the genomic locations that can be targeted by exploiting this approach are limited. In addition, the implemented approach, due to deamination of cytosine, confers transition mutations. It has been shown that most of the transition mutations at third position of codons are generally ineffectual mutations. Hence, the development of a strategy that confers both transition and transversion mutations could be beneficial.

## Author contributions

The author confirms being the sole contributor of this work and approved it for publication.

### Conflict of interest statement

The author declares that the research was conducted in the absence of any commercial or financial relationships that could be construed as a potential conflict of interest.
